# Childhood Self-Control and Unemployment Throughout the Life Span: Evidence From Two British Cohort Studies

**DOI:** 10.1177/0956797615569001

**Published:** 2015-06

**Authors:** Michael Daly, Liam Delaney, Mark Egan, Roy F. Baumeister

**Affiliations:** 1 Behavioural Science Centre, University of Stirling; 2 UCD Geary Institute, University College Dublin; 3 Department of Psychology, Florida State University; 4 King Abdulaziz University

**Keywords:** personality, self-control, unemployment, economic recession, human capital, open data, open materials

## Abstract

The capacity for self-control may underlie successful labor-force entry and job retention, particularly in times of economic uncertainty. Analyzing unemployment data from two nationally representative British cohorts (*N* = 16,780), we found that low self-control in childhood was associated with the emergence and persistence of unemployment across four decades. On average, a 1-*SD* increase in self-control was associated with a reduction in the probability of unemployment of 1.4 percentage points after adjustment for intelligence, social class, and gender. From labor-market entry to middle age, individuals with low self-control experienced 1.6 times as many months of unemployment as those with high self-control. Analysis of monthly unemployment data before and during the 1980s recession showed that individuals with low self-control experienced the greatest increases in unemployment during the recession. Our results underscore the critical role of self-control in shaping life-span trajectories of occupational success and in affecting how macroeconomic conditions affect unemployment levels in the population.

Self-control is one of the most useful human capabilities and has important implications for career success. Challenging work environments require employees to successfully inhibit their impulses and control their emotional expression in order to meet deadlines and avoid potential conflicts with customers and colleagues. Self-control may also enable workers to resist conflicting but desirable activities (e.g., leisure activities or sleep), minimize distractions, and form adaptive routines, thus facilitating the completion of demanding tasks and management of substantial workloads (de Ridder, Lensvelt-Mulders, Finkenauer, Stok, & Baumeister, 2012; Ent, Baumeister, & Tice, 2015; Hofmann, Vohs, & Baumeister, 2012; Schmidt, Hupke, & Diestel, 2012). Indeed, hard work is almost synonymous with self-control, as workers need to exert effort today to achieve valuable future benefits in the form of paychecks, bonuses, and promotions (Kaur, Kremer, & Mullainathan, 2010).

The research we report here builds on an emerging psychological literature demonstrating a close relationship between self-control and work performance and other work-related outcomes, including income and occupational prestige (de Ridder et al., 2012; Moffitt et al., 2011; Schmidt et al., 2012). Given these findings, it is somewhat surprising that self-control has not yet been linked to unemployment, a substantial global problem with vast consequences for people’s welfare. Using longitudinal data from two ongoing studies of British cohorts, we examined the extent to which self-control during childhood predicts spells of unemployment and the total amount of time people are unemployed throughout their working lives. To test whether adverse economic conditions may amplify the influence of self-control, we tracked unemployment outcomes as the United Kingdom entered the early-1980s recession.

## Childhood Self-Control

Individual differences in temperament emerge in the first decade of life and can have a large effect on a diverse range of adult life outcomes, including labor-market success (Caspi, Wright, Moffitt, & Silva, 1998; Heckman, Stixrud, & Urzua, 2006). Although temperament has been conceptualized in numerous ways, there is a degree of commonality across existing theoretical models. In particular, young children show enduring behavioral tendencies in their activity levels, their sensitivity to sensory stimuli, the degree to which they express positive (e.g., eagerness, joy) and negative (e.g., fear, irritability) emotions, and their capacity for self-control (Zentner & Bates, 2008). We focus on self-control, a basic component of temperament that is often indexed by observer ratings of a child’s ability to pay attention, persist on tasks, and suppress inappropriate behaviors (Zentner & Bates, 2008).

The effortful self-governance that typifies self-control has been described in a broad set of interrelated ways across subfields of psychology. For instance, in temperament research, the terms *effortful control* and *self-control* are often used synonymously to indicate the ability to suppress a dominant response in order to allow a subdominant response to be performed. Effortful control is thought to emerge from the developmental improvements in attentional control over the first several years of life (Rothbart, Ellis, Rueda, & Posner, 2003). In neuropsychology, *inhibitory control* captures both response inhibition and interference control, or the capacity to control attention, ignore distracting stimuli, and inhibit unwanted thoughts and emotions (Diamond, 2013). In personality research, *self-regulation* overlaps considerably with inhibitory control but also captures the capacity to maintain optimal levels of arousal (Diamond, 2013). Inhibitory control is also considered to underlie effective self-control, enabling children to resist or ignore tempting or distracting stimuli and inhibit impulsive behavior.

The benefits of self-control in a work-related context are readily apparent. Self-control helps people to ignore distractions and to persevere on and complete demanding tasks (Diestel & Schmidt, 2012; Ent et al., 2015). Furthermore, self-control is thought to underlie the emergence of conscientiousness, which is the personality trait most closely linked to school and career success (Eisenberg, Duckworth, Spinrad, & Valiente, 2014; Roberts, Kuncel, Shiner, Caspi, & Goldberg, 2007). Emerging evidence suggests that the benefits of self-control begin to accrue early in childhood and persist into adult life. Using data from a cohort of more than 1,000 children from New Zealand, Moffitt et al. (2011) demonstrated that measures of childhood self-control taken at ages 3 through 11 were closely related to adult outcomes in areas as diverse as physical health, income, substance abuse, and criminal behavior. Although suggestive evidence exists, no research has directly examined whether childhood self-control fosters successful entry into the labor force and assists workers in avoiding unemployment throughout adulthood.

## Childhood Self-Control and Unemployment

Self-control seems to be a highly plausible mechanism for attaining and retaining employment. Academic success has already been linked to good self-control, presumably because it facilitates concentration on studies and resistance to distracting temptations (Duckworth & Seligman, 2005; Tangney, Baumeister, & Boone, 2004). Self-control is also potentially valuable during the job-search process, which can be arduous and time-consuming. Individuals with lower self-control may be more likely to succumb to tempting or distracting alternatives and disengage from their search sooner (Ent et al., 2015; Kanfer, Wanberg, & Kantrowitz, 2001). Finally, a person with high self-control who is already employed may draw on these reserves to meet deadlines, arrive punctually, tolerate difficult customers, and so on (Schmidt et al., 2012). In school and the workplace, the advantage will lie with people who are better able to inhibit a preference for leisure, concentrate on their work, and regulate their emotions in favor of their education or career goals.

We hypothesized that the importance of self-control for successful entry into employment and for job retention is particularly pronounced in times of economic recession. During such periods, the returns to self-control are potentially highest, as the effort that needs to be devoted to job search increases. Employers may place a greater emphasis on self-control through processes such as internships, which make it possible to explicitly assess key skills like time management, persistence, and task completion. Also, when managers need to select staff for dismissal during adverse economic conditions, it is likely that the most self-controlled staff who invest heavily in their work life will be retained.

In summary, we hypothesized that children with low self-control will be much more likely than others to experience unemployment throughout their adult life, particularly when macroeconomic conditions are unfavorable. To test this idea, we capitalized on two British studies that have collected comprehensive measures of childhood characteristics and labor-force participation during adulthood.

## Study 1

### Method

We first examined data from the nationally representative British Cohort Study (BCS), a study of children born in Britain in a single week in 1970. We estimated the probability of unemployment at individual waves when the cohort members were ages 21, 26, 30, 34, 38, and 42; sample sizes ranged from 759 (a 10% random sample was conducted at age 21 because of funding issues) to 5,377 cohort members. Detailed data on total number of months of unemployment were available from 1986 through 2008, when the cohort members were ages 16 to 38 (sample size of 6,675). All sample sizes were determined by retaining all participants for whom data on the outcome variables and independent variables were available. All unemployment data that were analyzed are reported here.

#### Measures

##### Childhood self-control

Self-control scores were derived from nine items of the Disorganised Activity subscale of the Child Developmental Behaviors questionnaire, which was administered when the children were 10 years old. This scale was designed for the BCS and consists of items drawn chiefly from the Conners Teachers Hyperactivity Rating Scale (Conners, 1969) and the Rutter Teacher Behavioral Scale B (Rutter, 1967). Each child’s teacher rated the degree to which each item represented the child using a visual analogue scale ranging from *not at all* to *a great deal*. The questions centrally gauged attentional control (e.g., “cannot concentrate on a particular task,” “pays attention in class,” and “easily distracted”) and perseverance (“shows perseverance,” “completes tasks,” and “fails to finish tasks”; for a complete list of the items, see Section 1.2 in the Supplemental Material available online). The control of attention is a fundamental, perhaps even defining, component of self-control and one of the most common ways in which self-control is measured in laboratory settings (e.g., Hagger, Wood, Stiff, & Chatzisarantis, 2010). Similarly, the degree to which participants exhibit perseverance on experimental tasks (e.g., read-aloud tasks, unsolvable tracing or anagram tasks) is frequently used as a measure of self-control (Hagger et al., 2010). We reverse-scored ratings as appropriate so that higher scores always meant better self-control and then created a composite self-control variable by averaging the ratings for these nine questions (*M* = 31.09, *SD* = 10.22; range: 1.44–47; Cronbach’s α = .92). We then standardized this variable to have a mean of 0 and standard deviation of 1.

We tested the convergent and discriminant validity of this self-control measure in two ways: (a) using available measures in the BCS data and (b) using contemporary data collected specifically for this purpose. To construct an alternative measure of self-control in the BCS, we identified six teacher-rated items gauging persistence (e.g., “Does the child show perseverance?” and “percentage of time interested in other tasks”) and attentional control (e.g., “How well does the child concentrate?” and “percentage of time daydreaming”). The observed internal reliability coefficient for this measure was .83. This scale demonstrated strong convergent validity with our main self-control measure derived from the Child Developmental Behaviors questionnaire (*r* = .86, *p* < .01). Furthermore, in additional analyses, we found that the strength of the association between childhood self-control and our unemployment outcome measures did not differ when this alternative measure was used instead of our main measure.

To test discriminant validity using alternative BCS measures, we examined correlations between our main self-control measure and the Neuroticism (e.g., “worried and anxious” and “behaves nervously”) and Extraversion-Introversion (e.g., “rather solitary” and “introverted”) subscales of the Child Developmental Behaviors questionnaire. Both subscales demonstrated satisfactory levels of internal consistency (Neuroticism: Cronbach’s α = .85; Extraversion-Introversion: Cronbach’s α = .67). Our main self-control measure was moderately correlated with these commonly assessed basic personality dimensions: neuroticism: *r* = −.38, *p* < .01; extraversion-introversion (high scores indicate greater introversion): *r* = −.44, *p* < .01. The percentage of variance that the main and alternative self-control measures had in common was 4 to 5 times the percentage of variance that our main self-control measure had in common with the measure of either neuroticism or extraversion-introversion. Details of these personality measures are provided in Section 2 of the Supplemental Material.

To collect new data to test the convergent and discriminant validity of our main self-control measure, we conducted an online study of 100 American parents of children ages 5 through 12. These parents, who were recruited through Amazon Mechanical Turk, rated the temperament and behavior of their children on the Disorganised Activity scale used in this study and two contemporary self-control measures: the Brief Self-Control Scale (BSCS; Tangney et al., 2004) and the Domain-Specific Impulsivity Scale (DSIS; Tsukayama, Duckworth, & Kim, 2013). All three scales demonstrated high reliability, Cronbach’s αs = .83–.89. Scores on the nine-item Disorganised Activity scale (Cronbach’s α = .88) correlated strongly with scores on the BSCS (*r* = .75, *p* < .01) and DSIS (*r* = .75, *p* < .01). Thus, this online study provided support for convergent validity of our main measure and commonly used measures of childhood self-control.

To gauge discriminant validity of our main measure of self-control with contemporary data, we wanted to use measures similar to those we used in our test with BCS data. Our online study therefore included items commonly used to measure childhood emotional and peer problems, which are likely to correspond broadly with neuroticism and extraversion-introversion; these items were taken from the Strengths and Difficulties Questionnaire (SDQ; Goodman, 1997). The main self-control measure exhibited a significantly weaker correlation with emotional problems (*r* = −.35, *p* < .01) and peer problems (*r* = −.40, *p* < .01) than with the contemporary measures of self-control. Thus, this test also provided evidence for discriminant validity. The common variance between our main self-control measure and the contemporary measures of self-control was 4 times the common variance between our main self-control measure and these measures of peer and emotional problems. Taken together, these analyses suggest a strong degree of convergent and discriminant validity for our main measure of self-control. Our analysis of the validation data is described in full in Section 3 of the Supplemental Material.

##### Childhood covariates

Intelligence has previously been shown to predict labor-market outcomes, including unemployment (e.g., Caspi et al., 1998), and was positively correlated with self-control in this study (*r* = .41, *p* < .01). Consequently, we included intelligence as a covariate to rule out the possibility that self-control may predict unemployment because individuals with better self-control tend to be more intelligent. Intelligence was measured at age 10 using the British Ability Scales, which was made up of two verbal subscales (Word Definitions and Word Similarities) and two nonverbal subscales (Digit-Span and Matrices; Elliott, Murray, & Pearson, 1978). Intelligence scores were standardized to a mean of 0 and standard deviation of 1. Parental social class was derived from the father’s occupation in 1970; scores ranged from I, for professional occupations, to V, for unskilled workers. Child gender was also included as a covariate. As a test of robustness, we adjusted for measures of childhood conduct problems (e.g., “often disobedient” and “has tantrums”) and hyperactivity (e.g., “restless” and “can’t settle”); these items are described in full in Section 4 of the Supplemental Material.

##### Unemployment

Our outcome variables were (a) unemployment at ages 21, 26, 30, 34, 38, and 42 and (b) total months of unemployment from 1986 through 2008. At each wave, being in any kind of employment was coded as 0, and being unemployed was coded as 1. Unemployment rates in the sample ranged from a maximum of 10.8% in 1991, when the cohort members were age 21, to a minimum of 2.1% in 2004, when the participants were age 34 (see Table 1). The total number of months of unemployment was calculated from data collected across multiple waves, from the age-16 to the age-38 assessment. This variable was highly clustered at the left end of the scale—76% of cohort members never reported being unemployed, 14% reported being unemployed for a total of 1 to 12 months, and the remaining 10% reported being unemployed for anywhere from 13 to 269 months.

**Table 1.** Descriptive Statistics for Study 1 (British Cohort Study): Characteristics of Participants at Each Assessment Wave and of Participants With Lifetime Unemployment Data

Characteristic	Assessment wave						Lifetime-unemployment sample (*n* = 6,675)
	Age 21 (*n* = 759)	Age 26 (*n* = 4,339)	Age 30 (*n* = 5,377)	Age 34 (*n* = 4,700)	Age 38 (*n* = 4,405)	Age 42 (*n* = 4,824)	
Unemployment^a^	10.8%	4.9%	3.4%	2.1%	2.3%	2.4%	5.3 months
Self-control^b^ (mean)	31.17 (10.00)	32.69 (9.74)	31.79 (10.00)	32.01 (9.85)	32.33 (9.85)	32.12 (9.87)	31.66 (9.99)
Intelligence^c^ (mean)	76.16 (13.13)	78.93 (13.61)	77.94 (13.86)	78.24 (13.77)	78.75 (13.51)	78.43 (13.69)	77.31 (13.94)
Female (%)	50.6	51.1	45.7	46.6	48.3	48.9	50.6
Social class^d^ (%)							
I	5.7	7.7	7.2	7.2	7.8	7.3	6.8
II	21.1	26.3	25.2	25.5	26.1	25.5	24.0
III	57.3	52.6	53.4	53.1	52.2	53.0	54.1
IV	12.0	10.8	11.1	11.2	11.2	11.4	11.8
V	3.9	2.6	3.1	3.0	2.7	2.8	3.3

Note: Standard deviations are given in parentheses. *Lifetime unemployment* refers to unemployment from age 16 through age 38.

a The table shows the percentage of participants who were unemployed at each wave and the total number of months of unemployment for participants in the lifetime-unemployment sample. ^b^Unstandardized self-control scores ranged from 1.44 to 47.0; higher scores indicate better self-control. ^c^Unstandardized intelligence scores ranged from 23 to 125; higher scores indicate higher intelligence. ^d^Social class was derived from the father’s occupation: I = professional occupations, II = managerial or technical occupations, III = skilled workers, IV = semiskilled workers, and V = unskilled workers.

#### Statistical methods

We specified Probit regressions to determine the probability of unemployment at ages 21, 26, 30, 34, 38, and 42 and computed marginal effects to estimate percentage-point differences in the probability of unemployment at each age (Long & Freese, 2014). We also specified a negative binomial model to estimate the total number of months of unemployment from age 16 through age 38 and estimated the predicted number of months of unemployment at three levels of self-control (low = score 1 *SD* below the mean, medium = mean score, high = score 1 *SD* above the mean) to represent these differences more intuitively (Long & Freese, 2014). A negative binomial model is appropriate for overdispersed count data; in our sample, the mean total number of months of unemployment was much lower than the variance, and a significant number of cohort members reported 0 months of unemployment, so this was an appropriate analytic method (see Sturman, 1999, for a discussion of the merits of this model compared with others when analyzing count data). This model also controlled for the number of months for which employment data were available for each cohort member, to account for the possibility that individuals with lower self-control may have been more likely to disengage from the survey over time. The formal specifications of the models were as follows:

$$\begin{array}{l} \mathrm{Model}\;1:\\ \mathrm{unemployment at age}{\left(21/26/30/34/38/42\right)}_{i}\;=\;\beta 0+\beta 1\mathrm{childhood self}-{\mathrm{control}}_{i}+\beta 2{\mathrm{gender}}_{i}+\;\beta 3{\mathrm{childhood intelligence}}_{i}+\beta 4{\mathrm{social class}}_{i}+\varepsilon_{i} \end{array}$$

$$\begin{array}{l} \mathrm{Model}\;2:\\ \mathrm{total months of unemployment at ages}16-38_{i}\;=\;\beta 0+\beta 1\mathrm{childhood self}-{\mathrm{control}}_{i}+\beta 2{\mathrm{gender}}_{i}+\;\beta 3{\mathrm{childhood intelligence}}_{i}+\beta 4{\mathrm{social class}}_{i}+\beta 5{\mathrm{months of employment data recorded}}_{i}+\varepsilon_{i} \end{array}$$

### Results

#### Descriptive statistics

Table 1 presents descriptive statistics for this study. On average, males had lower self-control scores (*M* = 29.54) than females (*M* = 33.63), *t*(6703) = −17.1, *p* < .0001, which supports the rationale for controlling for gender in our models. Better self-control correlated with higher intelligence (*r* = .41, *p* < .01) and to a lesser extent with higher social class (*r* = .14, *p* < .01); these results are in line with previous research (Moffitt et al., 2011). To examine the relationship between total unemployment from age 16 to age 38 and level of self-control, we divided participants into three groups: participants with low self-control (those with scores 1 *SD* below the mean and lower; 17% of the sample), participants with high self-control (those with scores 1 *SD* above the mean and higher; 19% of the sample), and participants with medium self-control (all others). We found that participants with low-self-control accumulated 2.8 times as many months of unemployment as those with high self-control over the 22-year period examined (low self-control: *M* = 9.36 months, *SD* = 27.30; medium self-control: *M* = 4.86 months, *SD* = 17.51; high self-control: *M* = 3.35 months, *SD* = 12.71).

#### Regressions

**Fig. 1.**
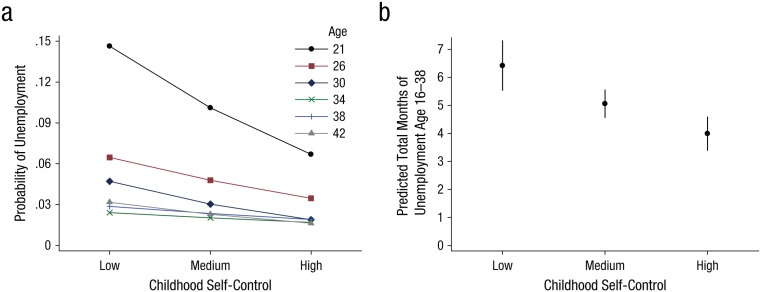
Results from Study 1: (a) predicted probability of unemployment at each assessment wave as a function of childhood self-control and (b) predicted marginal total number of months of unemployment as a function of childhood self-control. The error bars in (b) represent 95% confidence intervals. Low self-control = score 1 standard deviation below the mean; medium self-control = mean score; high self-control = score 1 standard deviation above the mean. Trends shown are adjusted for the inclusion of gender, intelligence, and social class in the regression equation.

Table 2 and Figure 1 present our main results. Controlling for gender, intelligence, and parental social class, we found that a 1-*SD* increase in childhood self-control was associated with the following reductions in the probability of unemployment in adulthood: 4.2 percentage points at age 21, 1.2 percentage points at age 26, 1.3 percentage points at age 30, and 0.6 percentage points at age 42. Self-control did not significantly predict unemployment at age 34 or age 38, when average unemployment rates were at their lowest. These results are shown graphically in Figure 1a. Across all six waves, a 1-*SD* increase in childhood self-control decreased the probability of unemployment by 1.3 percentage points on average; this was double the magnitude of the average effect of a 1-*SD* increase in intelligence (0.65 percentage points).

**Table 2.** Regression Results From Study 1 (British Cohort Study): Predicting Probability of Unemployment at Each Assessment Wave and Duration of Lifetime Unemployment

Predictor	Probability of unemployment						Lifetime unemployment (*n* = 6,675)
	Age 21 (*n* = 759)	Age 26 (*n* = 4,339)	Age 30 (*n* = 5,377)	Age 34 (*n* = 4,700)	Age 38 (*n* = 4,405)	Age 42 (*n* = 4,824)	
Self-control	−0.042** (0.012)	−0.012** (0.004)	−0.013** (0.003)	−0.002 (0.002)	−0.004 (0.002)	−0.006* (0.002)	−0.247** (0.055)
Intelligence	−0.003 (0.013)	−0.008* (0.004)	−0.007* (0.003)	−0.006* (0.002)	−0.007* (0.003)	−0.008** (0.003)	−0.200** (0.052)
Female gender	0.054* (0.023)	−0.033** (0.007)	−0.011* (0.005)	−0.005 (0.004)	−0.008 (0.005)	−0.013** (0.005)	−0.550** (0.099)
Social class							
II	−0.110* (0.055)	−0.005 (0.014)	−0.001 (0.011)	−0.017 (0.010)	−0.004 (0.010)	−0.001 (0.009)	−0.055 (0.205)
III	−0.019 (0.056)	0.000 (0.014)	0.004 (0.010)	−0.008 (0.010)	0.001 (0.009)	0.008 (0.009)	0.141 (0.194)
IV	−0.017 (0.064)	−0.002 (0.016)	0.009 (0.012)	−0.003 (0.012)	0.009 (0.012)	0.001 (0.010)	0.229 (0.231)
V	0.071 (0.092)	0.026 (0.027)	0.016 (0.018)	0.010 (0.019)	−0.016 (0.011)	0.034 (0.020)	0.812* (0.326)

Note: Standard errors are given in parentheses. *Lifetime unemployment* refers to unemployment from age 16 through age 38. For the probability of unemployment, the table presents marginal effects coefficients from Probit regressions. For the duration of lifetime unemployment, the table presents coefficients from a negative binomial model that controlled for the number of months of employment data recorded. Self-control and intelligence were standardized. Social class was derived from the father’s occupation: I = professional occupations, II = managerial or technical occupations, III = skilled workers, IV = semiskilled workers, and V = unskilled workers. Social class I was the reference group.

* *p* < .05. **p < .01.

Higher self-control was also significantly associated with less accumulated time spent unemployed from age 16 to age 38 (*b* = −0.247, *SE* = 0.055, *p* < .01). As shown in Figure 1b, the predicted number of months of unemployment was 6.34 (95% confidence interval, CI = [5.46, 7.22]), for participants with low self-control (1 *SD* below the mean), 4.99 (95% CI = [4.49, 5.47]) for those with mean self-control, and 3.91 (95% CI = [3.32, 4.51]) for those with high self-control (1 *SD* above the mean). Thus, our analyses indicated that from youth to age 38, participants with low self-control experienced 1.6 times as many months of unemployment as those with high self-control.

We conducted a robustness test of the association between self-control and unemployment by including control variables for mother-rated conduct problems and hyperactivity in our analyses. Given that these constructs overlap conceptually with self-control (e.g., Barkley, 1997) and that childhood traits such as aggressiveness have been shown to predict unemployment (e.g., Kokko, Bergman, & Pulkkinen, 2003), including conduct problem and hyperactivity was an attempt to stringently isolate the specific contribution of the self-control measure. Including these controls reduced the coefficients for self-control as a predictor of the probability of unemployment by an average of 13% across the individual time points and reduced the coefficient for self-control as a predictor of the duration of unemployment by 1% without affecting the significance levels of those coefficients. These results are detailed in full in Section 4 of the Supplemental Material.

## Study 2

### Method

Having demonstrated a link between childhood self-control and unemployment in Study 1, we used data from the British National Child Development Study (NCDS) to test the robustness of this association from 1974 through 2008. The NCDS contains extremely rich information on childhood characteristics, and thus allowed us to markedly extend the set of potentially confounding variables we considered in Study 1. Additionally, the recording of monthly data on labor-force status from age 16 through age 23 in the NCDS allowed us to test whether the potential impact of self-control on unemployment was amplified during the early 1980s, when the United Kingdom experienced an economic recession.

The NCDS is an ongoing longitudinal study following an initial cohort of 17,638 people born in Britain from March 3 through March 9, 1958. Extensive measures of participants’ early childhood environments were elicited through parental questionnaires, along with comprehensive measures of childhood characteristics elicited through teacher- and self-report instruments. To date, there have been eight follow-up waves (three in childhood, at ages 7, 11, and 16, and five in adulthood, at ages 23, 33, 42, 46, and 50). The attrition rate has been low: The number of cohort members responding in the last wave (in 2008) was 12,316. Hawkes and Plewis (2006) showed that those who have left the survey do not differ from the remaining participants in observable socioeconomic characteristics, which reduces the risk that participants with an elevated probability of unemployment are absent from our analyses. We examined unemployment using one wave from each decade of participants’ working lives. The final sample sizes in our analyses estimating the probability of unemployment at ages 23, 33, 42, and 50 ranged from 6,251 to 7,616 cohort members, and data on the total duration of unemployment from age 16 through age 50 were available for 10,107 participants. All sample sizes were determined by retaining all participants for whom data on the outcome variables and independent variables were available.

#### Measures

##### Childhood self-control

Childhood self-control was gauged at the first and second NCDS follow-ups, when participants were ages 7 and 11. At both of these waves, teachers rated the children’s behavior using the Bristol Social Adjustment Guide (Stott, 1969), which included a 13-item scale related to behavior considered “impulsive acting out without regard for consequences.” Research from this period found that children with low scores on this measure demonstrated poor educational performance (Chazan, 1968). Today, a wide array of measures are available for gauging individual differences in temperament early in life (Zentner & Bates, 2008). However, in 1965, when the NCDS participants were 7 years old, the evidence base supporting the measurement of individual differences in temperament in children was much less well developed (Kubzansky, Martin, & Buka, 2009).

Nonetheless, the self-control measure we used contains items comparable to those included in modern scales (e.g., Tsukayama et al., 2013), capturing individual differences in attentional control (e.g., “cannot attend or concentrate for long” and “too restless to remember for long”), persistence (“does not know what to do with himself, can never stick at anything long”), and impulsive behavior (“constantly needs petty correction”; for a complete list of the items, see Section 1.2 in the Supplemental Material). Teachers were asked to underline the phrases that they thought described the children’s behavior; each underlined item was scored as 1 point, and the maximum possible score was 13. We took the average of these scores at ages 7 and 11 to create a composite self-control measure (*M* = 11.61, *SD* = 1.67), coded so that a higher score was indicative of higher self-control. This variable was then standardized to have a mean of 0 and standard deviation of 1.

We built evidence for the validity of this measure using the data available in the NCDS and data we collected ourselves (including the contemporary self-control measures reported in Study 1). Although we could not estimate the convergent validity of the self-control measure in the NCDS data as we did in Study 1, we examined discriminant validity using a set of other measures also taken from the Bristol Social Adjustment Guide (Stott, 1969). To gauge emotional functioning, we used the 18-item Depression scale, and to assess extraversion-introversion, we combined scores on the 13-item Withdrawal scale (e.g., “quite cut off from people . . .” and “distant, shuns others’ company”) and the 18-item Unforthcomingness scale (e.g., “too shy to ask teacher’s help” and “says very little . . .”). (Details of these measures are provided in Section 2 of the Supplemental Material.) Our analyses supported discriminant validity of our self-control measure: It had a moderate negative association with depression (*r* = −.44, *p* < .01) and a weak negative correlation with extraversion-introversion (*r* = −.13, *p* < .01).

To test the convergent and discriminant validity of the main self-control measure using contemporary measures, we examined data collected in our online study of 100 parents of children ages 5 through 12 (see Study 1; also see Section 3 of the Supplemental Material). The 13-item scale that was our main self-control measure in Study 2 showed a high level of reliability (Cronbach’s α = .87), and scores on this scale correlated strongly with scores on the BSCS (Tangney et al., 2004), *r* = .74, *p* < .01, and DSIS (Tsukayama et al., 2013), *r* = .71, *p* < .01. Furthermore, the Study 2 self-control measure exhibited a weaker correlation with emotional problems (*r* = −.35, *p* < .01) and peer problems (*r* = −.38, *p* < .01) as gauged using the SDQ (Goodman, 1997), which provided evidence of discriminant validity. The percentage of common variance between the self-control measure utilized in this study and the contemporary measures of self-control was more than 4 times the common variance between the former measure and the SDQ measures of emotional and peer problems. We interpret the findings of these validation analyses as reasonably strong evidence in support of the convergent and discriminant validity of the self-control scale used in Study 2.

##### Childhood covariates

Childhood intelligence was assessed at age 11 using an 80-item general-ability test developed by the National Foundation for Educational Research in England and Wales (Pigeon, 1964). Parental social class was derived from the occupation of the father; scores ranged from I, for professional occupations, to V, for unskilled workers. Child gender was also included as a covariate. In our extended unemployment regressions, we included extensive controls for childhood variables that could plausibly have an impact on future employment trajectories. These variables included detailed measures of childhood health and family difficulties, as well as information on birth weight and region and household size at the time of the participant’s birth; their inclusion in the regressions did not significantly change the main results (see Section 5 in the Supplemental Material for further details). By adjusting our analyses for these factors, we aimed to rule out the possibility that self-control was acting as a proxy and that adverse experiences, childhood environmental conditions, or early health were the “true” causes of later unemployment. If including such variables in our regression model markedly diminished the link between self-control and unemployment, we would consider the relationship between self-control and unemployment to be affected by confounding.

As in Study 1, we also tested whether our results were robust to the inclusion of mother-rated measures that appeared to capture elements of conduct problems (e.g., “disobedient” and “fights other children”) and hyperactivity (“restless” and “squirmy”). These measures are described in full in Section 4 of the Supplemental Material.

##### Unemployment

Our outcome variables were (a) unemployment at ages 23, 33, 42, and 50 and (b) total number of months of unemployment from 1974 through 2008. For the wave measures of unemployment, being in any kind of full- or part-time employment was coded as 0, and being unemployed was coded as 1. Unemployment ranged from a peak of 10.9% at the age-23 wave to a low of 2.4% at the age-42 wave (see Table 3). We created a continuous variable gauging the total time each participant was unemployed by summing the number of months of unemployment from age 16 through age 50, using data collected across multiple study waves. As in Study 1, there was significant clustering at the left end of the scale—61% of the sample never reported any unemployment, 24% reported 1 to 12 months of unemployment, and the remaining 15% reported 13 to 341 months.

**Table 3.** Descriptive Statistics for Study 2 (National Child Development Study): Characteristics of Participants at Each Assessment Wave and of Participants With Lifetime Unemployment Data

Characteristic	Assessment wave				Lifetime-unemployment sample (*n* = 10,107)
	Age 23 (*n* = 7,616)	Age 33 (*n* = 6,938)	Age 42 (*n* = 7,247)	Age 50 (*n* = 6,251)	
Unemployment^a^	10.9%	4.8%	2.4%	2.8%	8.6 months
Self-control^b^ (mean)	11.67 (1.63)	11.69 (1.59)	11.75 (1.56)	11.79 (1.53)	11.77 (1.62)
Intelligence^c^ (mean)	44.56 (15.63)	45.03 (15.33)	45.37 (15.20)	46.25 (14.50)	44.06 (15.75)
Female (%)	43.4	43	47.1	48.1	50.1
Social class^d^ (%)					
I	4.2	4.4	4.3	4.6	4.1
II	14.0	13.9	13.9	14.6	13.2
III	61.2	61.8	61.7	61.6	61.6
IV	12.1	11.8	11.9	11.6	12.1
V	8.5	8.1	8.2	7.6	9.0

Note: Standard deviations are given in parentheses. *Lifetime unemployment* refers to unemployment from age 16 to age 50.

a The table shows the percentage of participants who were unemployed at each wave and the total number of months of unemployment for participants in the lifetime-unemployment sample. ^b^Unstandardized self-control scores ranged from 0 to 10.5; higher scores indicate better self-control. ^c^Unstandardized intelligence scores ranged from 0 to 80; higher scores indicate higher intelligence. ^d^Social class was derived from the father’s occupation: I = professional occupations, II = managerial or technical occupations, III = skilled workers, IV = semiskilled workers, and V = unskilled workers.

#### Statistical methods

We specified Probit regressions to estimate the probability of unemployment at ages 23, 33, 42, and 50 (Model 3) and a negative binomial model to estimate overall duration of unemployment from age 16 through age 50 (Model 4). (As in Study 1, a negative binomial model was appropriate because the mean total number of months of unemployment was much lower than the variance, and a significant number of cohort members reported no unemployment.) As in Study 1, we complemented the latter analysis with estimates of the predicted number of months of unemployment at three levels of self-control. We included controls for gender, intelligence, and parental social class in all regressions. For the model examining accumulated duration of unemployment (Model 4), we also included a continuous variable measuring the length of employment data available. The formal specifications of the models were as follows:

$$\begin{array}{l} \mathrm{Model}\;3:\\ \mathrm{unemployment at age}{\left(23/33/42/50\right)}_{i}\;=\;\beta 0+\beta 1\mathrm{childhood self}-{\mathrm{control}}_{i}+\beta 2{\mathrm{gender}}_{i}+\;\beta 3{\mathrm{childhood intelligence}}_{i}+\beta 4{\mathrm{social class}}_{i}+\varepsilon_{i} \end{array}$$

$$\begin{array}{l} \mathrm{Model}\;4:\\ \mathrm{total months of unemployment at ages}16-50_{i}\;=\;\beta 0+\beta 1\mathrm{childhood self}-{\mathrm{control}}_{i}+\beta 2{\mathrm{gender}}_{i}+\;\beta 3{\mathrm{childhood intelligence}}_{i}+\beta 4{\mathrm{social class}}_{i}+\beta 5{\mathrm{months of employment data recorded}}_{i}+\varepsilon_{i} \end{array}$$

As already noted, to test the robustness of our results, we repeated all analyses examining the association between self-control and unemployment adjusting for an additional array of childhood covariates (see Section 5 in the Supplemental Material). As in Study 1, we also tested the robustness of our findings to adjustment for conduct and hyperactivity problems (see Section 4 in the Supplemental Material).

If self-control-related differences in the unemployment rate were amplified as a result of economic recession, this would have implications for future investigative strategies, and possibly also for public policy. To test the effect of recession on the association between self-control and unemployment, we used monthly employment data collected on each participant from 1974 through 1982. The United Kingdom entered a recession in January 1980 (Jenkins, 2010), and the effect of this downturn on the labor market was dramatic; more than 619,000 jobs were lost, and the unemployment rate did not return to its prerecession level for 8 years. We created a recession variable that was coded as follows: 0 = June 1974–December 1979 (when cohort members were ages 16–21) and 1 = January 1980–February 1982 (ages 21–23). Using a Probit difference-in-difference model (Model 5) with clustered standard errors to account for nonindependence in repeated observations on the same individuals, we then estimated the average predicted probability of unemployment for individuals at different levels of self-control before and after the recession, using the margins command in Stata (Long & Freese, 2014). This model tested whether the average difference in unemployment level between participants with low and high self-control grew in the postrecession period. We entered the self-control and recession variables simultaneously with the Self-Control × Recession interaction term, in line with recommended practice (Aiken & West, 1991). We also included a monthly time trend. The formal specification of the model was as follows (the *t* subscript refers to time):

$$\begin{array}{l} \mathrm{Model}\;5:\\ \mathrm{monthly unemployment at ages}16-23_{\mathrm{it}}\;=\;\beta 0+\beta 1\mathrm{childhood self}-{\mathrm{control}}_{i}+\beta 2{\mathrm{gender}}_{i}+\;\beta 3{\mathrm{childhood intelligence}}_{i}+\beta 4{\mathrm{social class}}_{i}+\beta 5{\mathrm{month}}_{t}+\beta 6{\mathrm{recession}}_{t}+\beta 7\mathrm{childhood self}-{\mathrm{control}}_{i}*{\mathrm{recession}}_{t}+\varepsilon_{\mathrm{it}} \end{array}$$

### Results

#### Descriptive statistics

Table 3 presents descriptive statistics for this study. As in Study 1, males had lower self-control scores (*M* = 11.23) on average than females (*M* = 12.09), *t*(10105) = −27.7, *p* < .0001, and better self-control correlated with higher intelligence (*r* = .39, *p* < .01) and to a lesser extent with higher parental social class (*r* = .13, *p* < .01). Also as in Study 1, we divided participants into three self-control groups: participants with low self-control (those with scores 1 *SD* below the mean and lower; 13% of the sample), participants with high self-control (those scoring the maximum value of 0.83 *SD* above the mean; 28% of the sample), and participants with medium self-control (all others). We found that participants with low self-control accumulated around 3.3 times as many months of unemployment as those with high self-control (low self-control: *M* = 17.70 months, *SD* = 39.19; medium self-control: *M* = 8.13 months, *SD* = 24.54; high self-control: *M* = 5.42 months, *SD* = 16.95).

#### Main regressions

**Fig. 2.**
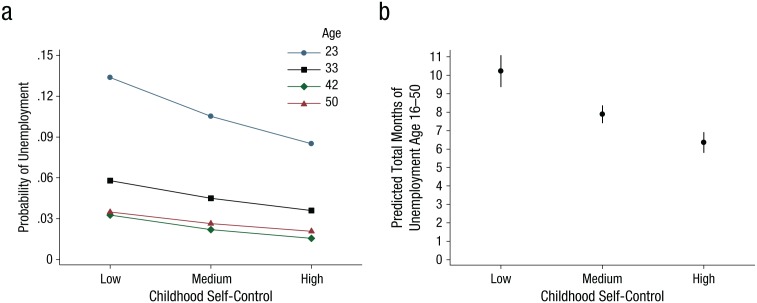
Results from Study 2 (National Child Development Study): (a) predicted probability of unemployment at each wave as a function of childhood self-control and (b) predicted marginal total number of months of unemployment as a function of childhood self-control. The error bars in (b) represent 95% confidence intervals. Low self-control = score 1 standard deviation below the mean; medium self-control = mean score; high self-control = score 0.83 standard deviations above the mean. Trends shown are adjusted for the inclusion of gender, intelligence, and social class in the regression equation.

Table 4 and Figure 2 describe our results. After controlling for gender, intelligence, and parental social class, we found that a 1-*SD* increase in childhood self-control predicted the following decreases in the probability of unemployment in adulthood: 2.6 percentage points at age 23, 1.2 percentage points at age 33, 0.9 percentage points at age 42, and 0.8 percentage points at age 50. These results are shown graphically in Figure 2a. On average, across the four waves examined, a 1-*SD* increase in self-control was associated with a reduction of 1.4 percentage points in the probability of unemployment.

**Table 4.** Regression Results From Study 2 (National Child Development Study): Predicting Probability of Unemployment at Each Assessment Wave and Duration of Lifetime Unemployment

Predictor	Probability of unemployment				Lifetime unemployment (*n* = 10,107)
	Age 23 (*n* = 7,616)	Age 33 (*n* = 6,938)	Age 42 (*n* = 7,247)	Age 50 (*n* = 6,251)	
Self-control	−0.026** (0.004)	−0.012** (0.002)	−0.009** (0.002)	−0.008** (0.002)	−0.261** (0.034)
Intelligence	−0.028** (0.004)	−0.021** (0.003)	−0.005** (0.002)	−0.013** (0.002)	−0.250** (0.030)
Female gender	0.002 (0.008)	−0.019** (0.006)	−0.003 (0.004)	−0.009* (0.004)	−0.388** (0.059)
Social class					
II	−0.032 (0.021)	−0.011 (0.016)	0.003 (0.009)	−0.003 (0.013)	−0.142 (0.158)
III	−0.016 (0.020)	−0.006 (0.014)	0.010 (0.008)	−0.005 (0.012)	−0.047 (0.144)
IV	−0.007 (0.022)	−0.013 (0.016)	0.008 (0.009)	−0.010 (0.013)	0.051 (0.162)
V	0.042 (0.024)	0.025 (0.018)	0.024* (0.011)	−0.003 (0.014)	0.356* (0.169)

Note: Standard errors are given in parentheses. *Lifetime unemployment* refers to unemployment from age 16 to age 50. For the probability of unemployment, the table presents marginal effects coefficients from Probit regressions. For the duration of lifetime unemployment, the table presents coefficients from a negative binomial model that controlled for the number of months of employment data recorded. Self-control and intelligence were standardized. Social class was derived from the father’s occupation: I = professional occupations, II = managerial or technical occupations, III = skilled workers, IV = semiskilled workers, and V = unskilled workers. Social Class I was the reference group.

* *p* < .05. **p < .01.

Our analysis of the total cumulative duration of unemployment showed that more self-controlled children went on to spend less time unemployed over their working lives (*b* = −0.261, *SE* = 0.034, *p* < .01). As shown in Figure 2b, the predicted number of months of unemployment was 10.30 (95% CI = [9.43, 11.16]) for participants with low self-control (1 *SD* below the mean), 7.93 (95% CI = [7.46, 8.41]) for those with mean self-control, and 6.39 (95% CI = [5.84, 6.94]) for those with high self-control (0.83 *SD* above the mean). As in Study 1, our analyses showed that from adolescence to midlife, participants with low self-control experienced 1.6 times as many months of unemployment as those with high self-control.

In our analysis including the extended range of important early-life controls (see Table S5 in the Supplemental Material), we found that the self-control coefficients remained significant at all time points except age 50. Thus, we can say that the association between self-control measured at ages 7 and 11 and unemployment throughout adulthood appears to be independent of key potentially confounding variables concerning childhood physical and mental health, region of birth, ethnicity, and family structure.

As in Study 1, we conducted a robustness check by using mother-rated measures assessing elements of conduct problems and hyperactivity. The inclusion of these variables reduced the coefficients for self-control as a predictor of the probability of unemployment by 7.6% on average across the individual time points and led to a 7% increase in the coefficient for self-control as a predictor of the duration of unemployment, without altering the significance levels of these coefficients. These measures and results are described in Section 4 of the Supplemental Material.

#### Self-control and unemployment before and after the recession

**Fig. 3.**
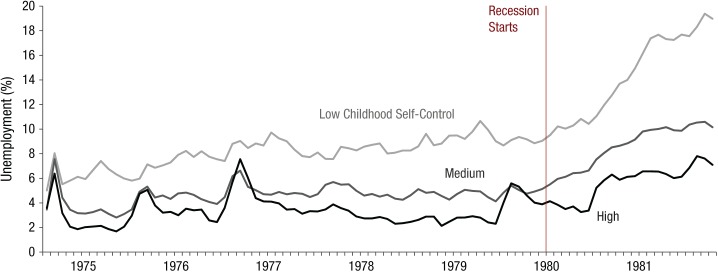
Descriptive unemployment statistics from Study 2 (National Child Development Study). Monthly data for August 1974 through October 1981 are shown for participants at three levels of childhood self-control (low = 1 *SD* below the mean or lower; high = 0.83 *SD* above the mean or higher; medium = all others).

**Fig. 4.**
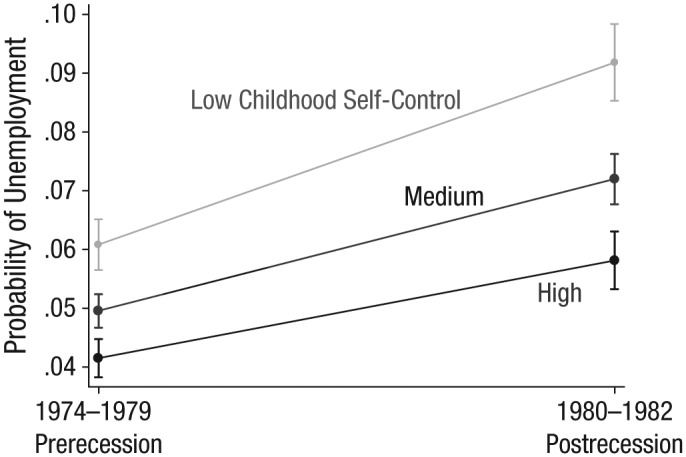
Results from Study 2 (National Child Development Study): predicted probability of unemployment before and after the 1980 recession, for participants at three levels of childhood self-control (low = 1 *SD* below the mean; medium = mean; high = 0.83 *SD* above the mean). Error bars represent 95% confidence intervals.

Unemployment levels rose sharply among participants with low self-control (1 *SD* below the mean and lower) in the aftermath of the 1980 recession (see Fig. 3 for descriptive unemployment rates for participants with low, medium, and high self-control; see Fig. 4 for predicted unemployment probabilities in the pre- and postrecession periods). From 1974 through 1979, the average predicted probability of unemployment for participants with low self-control (those scoring 1 *SD* below the mean) was 6.1%, compared with 4.1% for participants with high self-control (0.83 *SD* above the mean, the highest level of self-control reported). These figures rose to 9.2% and 5.8%, respectively, in the 1980–1982 period. The difference in the average unemployment level between the participants with low and high self-control therefore rose by 1.4 percentage points, from a 2-point gap to a 3.4-point gap,^1^ after controlling for covariates, as shown in Figure 4 (also see Table 5 for a summary of these analyses). Thus, the difference-in-difference analysis indicated that participants in the low-self-control group were disproportionately more likely to become unemployed after the onset of the recession. There was a similar gap between participants with low self-control and those with medium (mean) self-control, but it was smaller in magnitude (0.9 percentage points; see Fig. 4 and Table 5).

**Table 5.** Regression Results From Study 2 (National Child Development Study): The Effect of Childhood Self-Control on the Probability of Unemployment Before and After the 1980s Recession (597,858 Observations)

Self-control	1974–1979 (prerecession)	1980–1982 (postrecession)	Difference (postrecession – prerecession)	Difference in difference (relative to low self-control)
Low	0.061 (0.002)	0.092 (0.003)	0.031 (0.003)	—
Medium	0.050 (0.001)	0.072 (0.002)	0.022 (0.002)	0.009 (0.002)**
High	0.041 (0.002)	0.058 (0.002)	0.017 (0.003)	0.014 (0.003)**

Note: The table presents predicted probabilities calculated after a Probit regression, clustered by individual to account for nonindependence of repeated observations. Robust standard errors are given in parentheses. Gender, intelligence, social class, and a time trend were included in the analysis, but results for these predictors are not shown. Low self-control was defined as scoring 1 standard deviation below the mean on the standardized self-control measure, medium self-control was defined as scoring at the mean on this measure, and high self-control was defined as scoring at the maximum of 0.83 standard deviations above the mean.

** *p* < .01.

## Discussion

Our findings link childhood self-control to unemployment across adulthood. We utilized two large-scale prospective birth-cohort studies with detailed measurements of childhood psychological characteristics and comprehensive unemployment data gathered over four decades. Low childhood self-control predicted unemployment in adulthood, even decades later at age 50. The predictive strength of differences in childhood self-control was equal to or greater than that of intelligence, and childhood self-control was still a significant predictor after we controlled for variation in intelligence, social class, and an extensive range of family and health factors.

In Study 1, we found that children with lower self-control at age 10 experienced a higher cumulative duration of unemployment by age 38 and were more likely to be unemployed at ages 21, 26, 30, and 42 years. In Study 2, self-control was rated at ages 7 and 11, and lower scores predicted a greater duration of accumulated unemployment by age 50 and higher unemployment at ages 23, 33, 42, and 50. The link between self-control and unemployment peaked in magnitude when participants were in their early 20s. At that time, a 1-*SD* increase in self-control predicted an increase of more than 3 percentage points in the probability of unemployment across the two studies. However, even in the fourth and fifth decades of life, when unemployment rates in the cohorts were low (i.e., 2.4–2.8%), a 1-*SD* increase in self-control was associated with a large and statistically significant decrease in unemployment levels (~1 percentage point).

These findings contribute to a growing body of work suggesting that poor self-control is often a stable aspect of personality—and one that brings a host of long-run disadvantages (e.g., Moffitt et al., 2011; Slutske, Moffitt, Poulton, & Caspi, 2012). In Study 1, teachers rated children on whether they paid attention or were easily distracted and on whether they completed or gave up on tasks; in Study 2, teachers rated children on carelessness, the tidiness of their work, level of concentration, restlessness, posture, reliability, and rule breaking. It is perhaps understandable that adults who break rules, fail to complete tasks, lack concentration, and are careless and sloppy find fewer employment opportunities than their counterparts who follow rules, complete tasks, pay attention, and are careful workers. But the fact that these traits are sufficiently evident in young children to predict large, statistically significant and meaningful differences in employment among middle-aged adults indicates a remarkable degree of stability.

Although the contribution of self-control to unemployment showed substantial consistency over time in both cohorts, we hypothesized that this effect would become more pronounced in exceptionally poor macroeconomic circumstances, such as during a major recession. To test this idea, we examined monthly unemployment data for the NCDS cohort, who were tracked before and throughout the 1980s recession. We found a rapid growth in unemployment among participants with low self-control as the economy worsened in 1980 (see Figs. 3 and 4). The employment prospects of workers low in self-control appeared to be particularly vulnerable to macroeconomic fluctuations, which suggests that in difficult economic times, when employers need to scale back, many people with poor self-control either lose jobs or fail to get new ones.

The delayed consequences of such a potential differential impact remain to be examined in further research, but it is not safe to assume that people with poor self-control go back to work as soon as the macroeconomic picture brightens. Temporary career interruptions can have lasting, even permanent consequences, such as if one moves off the path of advancement, if one’s skills become obsolete, or if one eventually settles in at a lower-quality job (e.g., Arulampalam, 2001). Periods of unemployment also increase opportunities for abandoning healthy habits of regular sleep schedules, nutritious eating, good hygiene, and sobriety (and such opportunities may especially attract people lacking self-control); unemployment may also increase vulnerability to stress. As a result, the unemployed may become even less likely to reenter employment (e.g., Daly & Delaney, 2013; Digdon & Howell, 2008; Henkel, 2011; Krueger & Mueller, 2012).

The current research is limited in several respects. Although we adjusted for an extensive array of variables, it is possible that unobserved factors, such as unmeasured aspects of the family environment or genetic differences, predispose children to both poor self-control and later unemployment. Future studies comparing the impact of differences in self-control between siblings or twins would assist in ruling out these factors as explanations of the association between low self-control and unemployment (e.g., Delaney & Smith, 2012; Moffitt et al., 2011). An additional limitation is that the self-control measures used in Studies 1 and 2 were not originally designed for that purpose, which raises the possibility of measurement error that could have attenuated the relationship between self-control and unemployment. However, we gathered data that provided empirical support for the validity of the scales used as measures of self-control: Both scales we used correlated above .7 with modern self-control scales (BSCS: Tangney et al., 2004; DSIS: Tsukayama et al., 2013). Incorporating observational and parent- and self-report measures would help ensure that future studies precisely identify the role of self-control. Although this was not possible in the current study, our extended regressions show that self-control is unlikely to have acted as a proxy for other childhood characteristics (e.g., cognitive ability, family background, physical and mental health).

However, despite these adjustments, it remains difficult to determine how precisely we isolated the relationship between self-control and unemployment. For instance, by controlling for a detailed index of cognitive ability, we may have underestimated the contribution of self-control, given that aspects of cognitive ability, particularly working memory capacity, have been proposed to overlap with and facilitate self-control. Working memory capacity may promote effective self-control by protecting against attentional capture by tempting or distracting stimuli and by enabling important goals and standards to be kept in mind or protected from interference (Hofmann, Schmeichel, & Baddeley, 2012). Conversely, by failing to adjust for potentially important constructs, such as “grit” (i.e., perseverance and passion for long-term goals), that overlap with self-control (Duckworth & Gross, 2014), we may have overestimated the contribution of self-control.

Although we observed an enhanced contribution of self-control during a recession, it is unclear whether these striking findings are generalizable to other time periods and countries. The NCDS cohort’s early labor-market experience (at the end of the 1970s) occurred during a period of economic and industrial upheaval in the United Kingdom. Identifying whether an enhanced risk of unemployment among the less self-disciplined has occurred in other cohorts, such as those entering the labor market during the recent 2008 recession, will provide a new lens for understanding the effects of business-cycle fluctuations on both short- and long-run outcomes.

In summary, the present investigation provides robust evidence that poorer trait self-control is associated with higher unemployment across the life span. Teachers’ ratings of differences in self-control among children as young as 7 predicted unemployment more than four decades later. The policy implications are considerable: Improving children’s self-control could yield lifelong benefits to these individuals themselves, by raising their standard of living and reducing their danger of being unemployed, and also to broader society, by increasing employment and productivity. Emerging evidence suggests that the capacity for self-control is, to a certain degree, malleable and may be enhanced through training and sustained practice. Self-control and closely related traits have been shown to be enhanced by preschool programs, elementary-school interventions, and activities such as yoga or martial arts, computerized training games, and walking meditation exercises (Alan & Ertac, 2014; Diamond, 2012; Diamond & Lee, 2012; Heckman, Pinto, & Savelyev, 2013; Posner, Rothbart, & Tang, 2013). The present findings demonstrate the long-range power of self-control in predicting success in life and single out self-control as a key target for early intervention programs. Being able to regulate one’s behavior to comply with rules and systems during childhood appears to be a highly adaptive trait for engaging successfully in working life as an adult in a complex modern society.

## Supplementary Material

Supplementary material

## Supplementary Material

Supplementary material

## Notes

1. Difference-in-difference coefficients and standard errors were calculated using the lincom command in Stata.
