# Supplementary material for: Childhood Self-Control and Unemployment Throughout the Life Span: Evidence From Two British Cohort Studies
Source: Psychol Sci. 2015 Jun;26(6):709–23. doi: 10.1177/0956797615569001 (PMC4512256; doi:10.1177/0956797615569001)
Supplement: Supplementary material [file DS_10.11770956797615569001_SupplementalMaterial.pdf]

DOI: 10.1177/0956797615569001

Supplementary Information for:

Childhood self-control and unemployment throughout the lifespan: evidence from two British cohort studies.

Authors: Michael Daly*[1,2], Liam Delaney[1,2], Mark Egan[1], Roy F. Baumeister[3,4]

Affiliations:

[1] Behavioural Science Centre, Stirling Management School, Stirling University, FK94LA, United Kingdom.

[2] UCD Geary Institute, University College Dublin, Belfield, Dublin 4, Ireland.

[3] Florida State University, Tallahassee, Florida, United States,

[4] King Abdulaziz University, Saudi Arabia.

* To whom correspondence should be addressed. Email: michael.daly@stir.ac.uk

Section 1: Details of data-sets & self-control measurements used in Study 1 and Study 2.

Section 2: Convergent and discriminant validity in Study 1 and Study 2.

Section 3: Details of validation data and analyses comparing self-control scales in Study 1 and Study 2 with contemporary self-control scales.

Section 4: Unemployment regressions controlling for conduct problems and hyperactivity in Study 1 and Study 2.

Section 5: Unemployment regressions using extended controls in Study 2.

Section 6: Unemployment regressions controlling for prior unemployment in Study 1 and Study 2.

DOI: 10.1177/0956797615569001

Section 1: Details of data-sets & self-control measurements used in Study 1 and Study 2.

1.1 Data-sets Used

British Cohort Study (Study 1) (accessed October 4th 2014)

1. Activity Histories, 1986-2000 (BCS 6943).
2. Ten-Year Follow-up, 1980 (BCS 3723).
3. Sixteen-Year Follow-up, 1986 (BCS 3535).
4. Twenty-One Year Sample-survey, 1992 (BCS 4715).
5. Twenty-Six Year Follow-up, 1996 (BCS 3833).
6. Thirty Year Follow-up, 2000 (BCS 5558)
7. Thirty-Four Year Follow-up, 2004-05 (BCS 5585).
8. Thirty-Eight Year Follow-up, 2008-09 (BCS 6557).
9. Forty-Two Year Follow-up, 2012-2013 (BCS 7473).

National Childhood Development Study (Study 2) (accessed October 4th 2014)

1. NCDS Activity Histories, 1974-2008 (NCDS 6942).
2. NCDS Childhood Data, Sweeps 0-3, 1958-1974 (NCDS 5565).
3. NCDS Sweep 4 data (NCDS 5566).
4. NCDS5 Cohort Member Interview and booklets data (NCDS 5567).
5. NCDS Sweep 6 data (NCDS 5578).
6. NCDS8 data labels library for SAS version (NCDS 6137).

All data-sets were downloaded from the UK Data Service:
http://discover.ukdataservice.ac.uk/series/?sn=2000032

DOI: 10.1177/0956797615569001

1.2 Details of Self-Control Measurements

British Cohort Study (Study 1)

Self-control scores were derived from teacher ratings of the 11-item Disorganised Activity subscale of the 53-item Child Developmental Behaviors questionnaire, taken when the child was 10 years old. This measure was designed for the British Cohort Study and consists of items drawn chiefly from the Conners Teachers Hyperactivity Rating Scale (Conners, 1969) and the Rutter Teacher Behavioral Scale B (Rutter, 1967). We omitted two items due to poor face validity ("Confused or hesitant", "Shows lethargic/listless behaviour"). Dropping these two items reduced the reliability of the scale from 0.93 to 0.92 and had essentially no effect on the self-control coefficients in the regressions. The questions used are listed below:

1. Child is daydreaming. **R**
2. Cannot concentrate on particular task. **R**
3. Becomes bored during class. **R**
4. Shows perseverance.
5. Easily distracted. **R**
6. Pays attention in class.
7. Forget on complex tasks. **R**
8. Completes tasks.
9. Fails to finish tasks. **R**

R means the score was reverse coded. All these scores were then summed and averaged to create a composite self-control score, which was then standardized to have a mean of 0 and standard deviation of 1.

National Childhood Development Study (Study 2)

Childhood self-control was gauged as part of the first and second NCDS follow-ups at ages 7 and 11. At both of these ages teachers rated the child's behaviour using the British Social Adjustment Guide (Stott, 1969) which included a 13-item scale related to behaviour

considered "impulsive acting out without regard for consequences". Teachers were asked to underline the phrases which applied to the child. The questions used are listed below:

1. Sometimes eager, sometimes doesn't bother (answering questions).
2. Constantly needs petty correction (classroom behaviour).
3. Too restless to remember for long (effect of correction).
4. Cannot attend or concentrate for long (cannot sit still when read to or during broadcasts, plays with things under desk) (attentiveness).
5. Rough and ready, slapdash (standard) (manual).
6. In informal play starts off others in scrapping and rough play.
7. Does not know what to do with himself, can never stick at anything long (free activity).
8. Misbehaves when teacher is out of room (liking the limelight).
9. Careless, untidy, often loses or forgets books, pen (belongings).
10. Gets very dirty during day (care for appearance).
11. Slumps, lolls about (posture).
12. Foolish pranks when with a gang (nuisance).
13. Follower in mischief (nuisance)

The NCDS does not contain an individual score breakdowns for these questions - instead there is only an aggregate score. We took the average of these two scores to create a composite self-control score where a higher score meant worse self-control. Scores ranged from 2.5-13 out of a possible maximum of 13. We then inverted these scores so that a higher score meant better self-control and standardized the variable to a mean of 0 and standard deviation of 1. Due to clustering on the scale, the maximum value of the standardized variable was 0.83 SD above the mean.

DOI: 10.1177/0956797615569001

Section 2: Convergent and discriminant validity in Study 1 and Study 2.

2.1 Details of Convergent and Discriminant Validity Scales

British Cohort Study (Study 1)

Convergent Validity

To test convergent validity with our self-control scale we used alternative teacher-rated measures which gauged persistence and attentional control. The children's scores on the below questions were coded so that a higher score always meant better self-control. Each of the six individual items was then standardized and the six items were summed. The summed variable itself was then standardized the create the Convergent Validity scale (Cronbach's α = 0.82).

1. How well does the child concentrate (score range 1-47)
2. Percentage of time concentrating (0-100)
3. Percentage of time fidgeting (0-95)
4. Does the child show perserverance (0-100)
5. Percentage of time interested in other tasks (0-100)
6. Percentage of time daydreaming (0-100)

This scale demonstrated strong convergent validity with our main self-control measure derived ($r = 0.86$, $p<0.01$).

Discriminant Validity

To test discriminant validity we constructed scales of Neuroticism and Introversion. As above, all questions were coded so that a higher score meant more neuroticism or more introversion. The scales were created by standardizing each variable individually, summing them and then standardizing the resultant variable. The neuroticism questions were:

1. Afraid of new things / situations (all scored 1-47)
2. Behaves 'nervously'

DOI: 10.1177/0956797615569001

3. Fussy or over-particular
4. Worried and anxious
5. Anxious, worried

All items were scored from 1-47. This scale demonstrated high internal consistency (Cronbach's $\alpha = 0.85$) and displayed moderate correlation with our main self-control measure ($r = -0.38$, $p<0.01$).

The questions measuring introversion were:

1. Rather solitary
2. Listless or lethargic
3. Sullen or sulky
4. Introverted

All items were scored from 1-47. This scale demonstrated moderate-high internal consistency (Cronbach's $\alpha = 0.67$) and was moderately correlated with our main self-control measure ($r = -0.44$, $p<0.01$).

National Child Development Study (Study 2)

Discriminant Validity

The NCDS data does not contain appropriate measures to estimate convergent validity. Instead we estimated discriminant validity using (i) an 18-item Depression scale assessing emotional functioning, measured at 7 and 11 years and (ii) an Introversion scale created using a combination of a 13-item Withdrawal scale and 18-item Unforthcomingness scale, both measured at 7 and 11 years. The discriminant validity scales were created by standardizing the constituent variables, summing them and then standardizing the resultant variable. For example to create our Introversion scale we standardized Unforthingcomingness at 7 years, Unforthcomingness at 11 years, Introversion at 7 years and Introversion at 11 years. We then summed these variables and standardized the result to create the Introversion scale.

The Depression scale questions were:

1. Depends on how he feels (asking teacher's help).
2. Varies noticeably from day to day (persistence in class work).
3. Sometimes alert, sometimes lethargic in team games.

DOI: 10.1177/0956797615569001

4. In free activity sometimes lacks interest.
5. Persistence in manual tasks varies greatly.
6. Impatient, loses temper with job (persistence - manual tasks).
7. Flies into a temper if provoked (physical prowess).
8. Can work alone but has no energy (persistence in class work).
9. Lacks physical energy (persistence manual tasks).
10. Has no life in him (class room behaviour).
11. Apathetic (just sits) (attentiveness).
12. Shuffles restlessly (posture).
13. In asking teacher's help too apathetic to bother.
14. Dull listless eyes.
15. Always sluggish, lethargic in team games.
16. Sometimes wanders off alone (companionship).
17. Speech is thick, mumbling, inaudible.
18. Expression is miserable, depressed (under the weather) seldom smiles.

Our self-control measure demonstrated a moderate negative association with the depression scale ($r = -0.44$, $p<0.01$).

The questions used to measure Unforthcomingness were:

1. Chats only when alone with teacher.
2. Bursts into tears (attitude to correction).
3. Never offers to help teacher with jobs by pleased when asked.
4. Submissive, takes less wanted position, a ball fetcher (team games).
5. Too timid to be naughty (class room behaviour).
6. Lies from timidity (truthfulness).
7. Likes sympathy but reluctant to ask.
8. Never brings flowers, gifts, although classmates often do.
9. Never brings objects he has found, drawings, models, etc, to show teacher although classmates often do.
10. Associates only with one other child and mostly ignores the rest.
11. Waits to be noticed before greeting teacher.
12. Never makes first approach (talking to teacher).
13. Too shy to ask teacher's help.

DOI: 10.1177/0956797615569001

14. When answering questions, gets nervous, blushes, cries when questioned
15. Shrinks from active play in informal play.
16. Mumbles shyly, awkwardly in response to greeting.
17. Can't get a word out of child (talking to teacher).
18. Says very little when talking to teacher.

The questions used to measure Withdrawal were:

1. Absolutely never greets teacher.
2. Does not answer when greeted.
3. Makes no friendly or eager response (general manner with teacher).
4. Avoids talking to teacher (distant, deep).
5. Dreamy and distracted (lives in another world) (attentiveness).
6. Distant and uninterested (persistence in manual tasks).
7. Dreamy, uninterested in team games.
8. Distant, shuns others' company.
9. Keeps clear of adults even when hurt or wronged (liking for sympathy).
10. Quite cut off from people, you can't get near him as a person (general with teacher).
11. Unresponsive eyes.
12. Speech is an incoherent rumbling chatter.
13. In contacts with teacher, is like a suspicious animal

The withdrawn and introversion scales were combined to create an Introversion scale (Cronbach's $\alpha = 0.64$) . Our self-control variable displayed a weak negative correlation with this Introversion scale ($r = -0.15$, $p<0.01$).

DOI: 10.1177/0956797615569001

Section 3: Details of validation data and analyses comparing self-control scales in Study 1 and Study 2 with contemporary self-control scales.

In order to test the validity of the main measurements used in Study 1 and 2, we conducted an online study to examine their correlation with 3 modern self-control scales. We recruited a sample of 100 American parents of children aged 5-12 via the website Amazon Mechanical Turk. Our survey was advertised as a “Adult and child personality study”.

The survey routed participants to complete a set of self-control scales if they reported having a child aged 5-12 or a set of adult personality scales (not described here) if they reported not having a child in that age range. If a parent had more than one child in the 5-12 age range, they were asked to complete the survey with respect to the oldest child (e.g. if they had a 10 year old and an 8 year old , they were instructed to complete the survey about the 10 year old).

Since participants received compensation for their participation regardless of which task they completed (rating their own or their child’s personality), this design minimized the risk that participants may claim to have a child in order to receive payment for completing the survey. There was no obvious pattern of deceit in the answers of either group; the participants took several minutes to complete the survey, indicating that they weren’t just answering as quickly as possible, there was no clustering of answers at particular points on the scale (e.g. participants may have just mindlessly clicked the right-most answer every time) and in line with the general findings of the literature, female children were reported to have better self-control than boys on average on all five self-control scales.

The sample of parents rated their child’s temperament and behaviour on the below five scales:

Cohort studies

(i) The 11-item scale in the British Cohort Study (BCS).
(ii) The 13-item ‘Inconsequential Behaviour’ scale from the National Child Development Study (NCDS).

Modern scales

DOI: 10.1177/0956797615569001

(iii) 10-items gauging peer and emotional problems drawn from the Strengths & Difficulties Questionnaire (Goodman, 1997)
(iv) The 13-item 'Brief Self-Control Scale' (Tangney et al., 2004)
(v) The 7-item Domain-Specific Impulsivity Scale (Tsukayama et al., 2013)

To test the convergent validity of the scales used to measure self-control in Study 1 and 2, we examined their correlation with contemporary self-control scales (iv) and (v). The Brief Self-Control Scale (BSCS) is one of the most used modern self-control scales and has been used extensively to gauge self-control in school-aged children (e.g. Duckworth & Seligman, 2005; Duckworth & Seligman, 2006). The Domain-Specific Impulsivity Scale (DSIS) is was developed to gauge self-control and has been validated against existing measures including the BSCS (Duckworth, Tsukayama, Kirby, 2013; Tsukayama, Duckworth, & Kim, 2013). To test the discriminant validity of the scales used in Study 1 and Study 2 we used 10 items from (v) the Strengths & Difficulties Questionnaire (SDQ) covering two facets; peer and emotional problems. If better self-control did not correlate strongly with more peer problems and emotional problems, this would be evidence of discriminant validity given that these represent different constructs.

In our sample of 100 parents, all self-control scales demonstrated high reliability with Cronbach's alpha scores ranging from 0.83 to 0.89. In addition to the full 11-item and 13-item measures used in the British Cohort Study and National Child Development Study respectively, we tested reduced versions of these scales: (i) a 9-item version of the scale used in the BCS which removed 2 items due to poor face validity ("lethargic/listess" and "hesitant") (ii) a 7-item version of the scale used in the NCDS which removed 6 items due to weak face validity (e.g. "rough and ready, slapdash", "follower in mischief"). Removing these items did not substantially alter the reliability scores of the scales nor the magnitude of the correlations with the modern self-control measures.

Correlations between old and new self-control scales

Table S1 shows the correlation results from the validation study. The 9-item self-control scale in Study 1 correlated with better self-control on the BSCS ($r=0.75$, $p<0.01$) and DSIS ($r=0.75$, $p<0.01$), indicating a strong level of convergent validity, and a much lesser extent with emotional ($r= -0.35$, $p<0.01$) and peer problems ($r= -0.40$, $p<0.01$) as measured by the SDQ, indicating evidence of discriminant validity. Similarly, the 13-item scale from Study 2 also correlated strongly with better self-control on the BSCS ($r=0.74$, $p<0.01$) and DSIS ($r=0.71$,

p<0.01) and exhibited a weaker correlation with emotional (r= -0.35, p<0.01) and peer problems (r= -0.40, p<0.01).

Table S1. Correlation matrix comparing the self-control scales used in Study 1 and Study 2 to the Brief Self-Control Scale (BSCS), the Domain-Specific Impulsivity Scale (DSIS) and Peer and Emotional Problems subscales of the Strengths & Difficulties Questionnaire (SDQ).

	**Study 1**[a]	**Study 2**[b]	**BSCS** [c]	**DSIS** [d]	**SDQ: Peer problems**[e]
**Study 2** [b]	0.74	1			
**BSCS** [c]	0.75	0.74	1		
**DSIS** [d]	0.75	0.71	0.61	1	
**SDQ: Peer problems** [e]	-0.40	-0.38	-0.32	-0.32	1
**SDQ: Emotional Problems** [e]	-0.35	-0.35	-0.38	-0.28	0.48

[a] Self-control scale from the British Cohort Study (Study 1).
[b] Self-control scale from the National Child Development Study (Study 2).
[c] Brief Self-Control Scale.
[d] Domain-Specific Impulsivity Scale.
[e] Subscale of the Strengths & Difficulties Questionnaire.
All correlations are significant at p<0.01

We interpret the findings of this validation study as reasonably strong evidence for the convergent and discriminant validity of the self-control scales used in Study 1 and 2. The two scales used in Study 1 and Study 2 show high levels of reliability with values approaching Cronbach's alpha of 0.9 and they are not sensitive to the removal of specific items or sets of items in terms of substantially reduced reliability nor in terms of convergent validity with the contemporary self-control scales. We identify similar levels of convergent validity with contemporary self-control measures as identified in the recent literature during the development of self-control measures (Tsukayama et al., 2013). Furthermore, we show that the two measures of self-control correlate strongly together and not with other commonly measured constructs such as peer and emotional problems, providing evidence of discriminant validity: the percentage of variance in common between the self-control

measures in Study 1 and 2 and the modern self-control measures was 4 times greater than the common variance between the self-control measures in Study 1 and 2 and the measures of peer and emotional problems.

We acknowledge two main limitations of the validation study. Firstly, the sample used in the validation study was a group of American parents rather than British teachers. Secondly, the former group rated these measures in 2014 whereas the latter rated them between 1965 and 1980. These changes introduce the potential for measurement bias, potentially due to changing perceptions of question meaning or different cultural standards to judge children against in terms of what constitutes ‘normal’ behaviour. Despite these issues, which cannot be avoided when comparing older scales against modern measures, we consider the evidence to on balance favor the use of the scales in Study 1 and 2 as good indicators of childhood self-control.

DOI: 10.1177/0956797615569001

Section 4: Unemployment regressions controlling for conduct problems and hyperactivity.

This section details the mother-rated measures we identified in Study 1 and 2 as capturing elements of conduct probolems and hyperactivity. Given that both these constructs are conceptually similar to self-control (Barkley, 1997), including these measures as control variables in our unemployment regressions represent a stringent attempt to isolate the contributing effects of self-control.

Table S2 describes the items used in both studies. Our selection of these measures was guided by attempting to match the items available in the data with those from the 'conduct problems' and 'hyperactivity' sections in the well-validated Strengths and Difficulties Questionnaire. The composite 'conduct problems' and 'hyperactivity' variables were created by taking the average of their component measures.

DOI: 10.1177/0956797615569001

Table S2. Conduct problems and hyperactivity in Study 1 and Study 2.

Variable	N	Mean	SD	Min	Max	α
Study 1 (age 10)						
Often disobedient		25.87	25.15	0	100	
Fights other children		17.73	20.09	0	100	
Bullies other children		13.79	14.36	0	99	
Often tells lies		18.86	20.32	0	100	
Takes others' belongings		12.87	14.72	0	100	
Has tantrums		17.74	14.72	0	100	
Destroys belongings		13.16	15.21	0	100	
**Conduct problems**	8,022	17.31	13.46	0	96.71	0.76
Restless		34.54	31.00	0	100	
Squirmy		27.97	28.36	0	100	
Can't settle		23.39	25.70	0	100	
Easily distracted		33.18	30.33	0	100	
Fails to finish things		27.80	27.36	0	100	
**Hyperactivity**	8,376	29.30	20.81	0	100	0.78
Study 2 (age 7 and 11)						
Disobedient		1.17	0.90	0	4	
Fights other children		1.19	0.95	0	4	
Generally destructive		0.26	0.62	0	4	
**Conduct problems**	10,065	0.87	0.61	0	4	0.56
Restless		0.47	0.88	0	4	
Squirmy		1.05	1.14	0	4	
Can't settle		0.81	1.03	0	4	
**Hyperactivity**	10,171	0.78	0.74	0	4	0.55

Better self-control correlated with fewer conduct problems in Study 1 ($r= -0.25$, $p<0.01$) and Study 2 ($r= -0.32$, $p<0.01$) and with less hyperactivity in Study 1 ($r= -0.35$, $p<0.01$) and Study 2 ($r= -0.48$, $p<0.01$).

Table S3 describe the results of including these variables in our unemployment regressions in Study 1. Exhibiting more conduct problems in childhood was associated with a higher probability of unemployment at age 26, 30 and 38. Conduct problems were also associated with a greater lifetime duration of unemployment. Hyperactivity displayed no significant relationship with later unemployment at any time-point. The inclusion of these two variables

DOI: 10.1177/0956797615569001

reduced the self-control coefficients by an average of 23 per cent for the individual timepoints and 1 per cent for predicting lifetime unemployment.

Table S3. Unemployment regressions in Study 1 controlling for childhood conduct problems and hyperactivity.

Outcome	Age 21[a]	Age 21[a]	Age 26[a]	Age 26[a]	Age 30[a]	Age 30[a]	Age 34[a]	Age 34[a]
Observations	779	698	4,432	4,075	5,484	5,030	4,792	4,419
Self-control[c]	-0.040***	-0.037***	-0.015***	-0.010***	-0.015***	-0.015***	-0.004	-0.003
	(0.012)	(0.011)	(0.004)	(0.003)	(0.003)	(0.003)	(0.002)	(0.002)
Conduct problems[c]		0.012		0.010***		0.005*		0.005**
		(0.012)		(0.003)		(0.003)		(0.002)
Hyperactivity[c]		-0.012		0.006*		-0.005*		-0.003
		(0.012)		(0.003)		(0.003)		(0.002)

Outcome	Age 38[a]	Age 38[a]	Age 42[a]	Age 42[a]	Age 16-38[b]	Age 16-38[b]	--	--
Observations	4,497	4,136	4,921	4,526	6,879	6,290	--	--
Self-control[c]	-0.005**	-0.002	-0.008***	-0.007***	-0.275***	-0.271***		
	(0.002)	(0.002)	(0.002)	(0.002)	(0.054)	(0.059)		
Conduct problems[c]		0.006**		0.001		0.170***		
		(0.002)		(0.002)		(0.063)		
Hyperactivity[c]		0.004		0.003		-0.043		
		(0.002)		(0.002)		(0.061)		

[a] Regressions contain Probit marginal effects coefficients.
[b] Regressions contain negative binomial coefficients.
[c] Self-control, conduct problems and hyperactivity are standardized.
Controlling for childhood intelligence, gender and parental social class.
Standard errors in parentheses
*** p<0.01, ** p<0.05

Table S4 describes the results of including these variables in our unemployment regressions in Study 2. Conduct problems were associated with a higher probability of unemployment at age 26 and 33 and a greater lifetime duration of unemployment. Hyperactivity was associated with a reduced probability of unemployment at age 23 and a reduced lifetime duration of unemployment. The inclusion of these variables reduced the self-control coefficients by 7.6 per cent on average for the individual timepoints and led to a 7 per cent increase in the self-control coefficient for predicting lifetime unemployment.

DOI: 10.1177/0956797615569001

We interpret these regressions as evidence that the self-control measurement is robust to controls for childhood hyperactivity and conduct problems in both studies.

DOI: 10.1177/0956797615569001

Table S4. Unemployment regressions in Study 2 controlling for childhood conduct problems and hyperactivity.

Observations	7,616	6,568	6,938	5,979	7,247	6,265
Outcome	Age 23 [a]	Age 23 [a]	Age 33 [a]	Age 33 [a]	Age 42 [a]	Age 42 [a]
Self-control[c]	-0.027***	-0.028***	-0.012***	-0.011***	-0.009***	-0.011***
	(0.003)	(0.003)	(0.002)	(0.002)	(0.002)	(0.002)
Conduct problems[c]		0.019***		0.012***		0.003
		(0.003)		(0.003)		(0.002)
Hyperactivity[c]		-0.014***		-0.005*		-0.004*
		(0.003)		(0.003)		(0.002)
Observations	6,251	5,407	10,107	8,650	--	--
Outcome	Age 50 [a]	Age 50 [a]	Age 16-50 [b]	Age 16-50 [b]	--	--
Self-control[c]	-0.008***	-0.006***	-0.261***	-0.279***		
	(0.002)	(0.002)	(0.034)	(0.039)		
Conduct problems[c]		0.003		0.118***		
		(0.002)		(0.035)		
Hyperactivity[c]		0.001		-0.104***		
		(0.002)		(0.037)		

[a] Regressions contain Probit marginal effects coefficients.
[b] Regressions contain negative binomial coefficients.
[c] Self-control, conduct problems and hyperactivity are standardized.
Controlling for childhood intelligence, gender and parental social class.
Standard errors in parentheses
*** p<0.01, ** p<0.05

Section 5: Unemployment regressions using extended controls in Study 2.

As a robustness check of our analysis of the association between childhood self-control and later unemployment, we included extensive childhood controls that could plausibly have affected future employment trajectories. Firstly we considered adverse childhood experiences and mental health. We created dummy variables for whether the childhood home had housing difficulties, financial difficulties, domestic tension, alcoholism, physical handicap, unemployment or sickness, mental illness or 'mental subnormality' and other serious difficulties. These 9 variables were then summed into a 'Family Difficulties' scale ranging from 0-6 where a higher score meant more difficulties. For health controls we included

dummies for whether the child had 'mental retardation' at age 7, low birth weight (less than 88 ounces), whether the child received psychiatric treatment by age 11 and/or was recorded as being emotionally maladjusted at age 7, and whether the child suffered frequent headaches, migraines or epilepsy at age 7. The rationale for controlling for these kinds of early childhood trauma and mental health conditions was that such early events can have significant impacts on adult socioeconomic outcomes, with research suggesting a negative impact on employment, education and earnings resulting from childhood abuse (Currie & Widom 2010), early mental-health disorders (Kawakami et al. 2012, Goodman, Joyce, & Smith, 2011) and other adverse childhood experiences (Liu et al. 2013).

Secondly we included demographic and regional controls. We created dummies for the 11 different regions of birth represented in the NCDS, a continuous variable for the husband's age at the time of the child's birth and for household size (continuous from 0-7 and 8+ thereafter). We created a dummy variable for race but since the vast majority of respondents identified as Euro-Caucasian (almost 96 per cent of those for whom data is available), we coded this as 0 and everything else as 1. There is information on the mother's marital status at the time of the child's birth, but we did not use this measure since over 99 per cent of the sample reported being married or in a stable union,

Table S5 describes our unemployment regressions with extended control variables. Self-control remains a significant predictor of unemployment at ages 23, 42 and 50, although the coefficient sizes are reduced. Other notable results include: (1) significant regional differences in predicting lifetime unemployment, with the East and South-East, South and South-West regions reporting less unemployment than the North; (2) Non-whites had a significantly higher probability of unemployment at ages 23 and 50; (3) Children who received psychiatric treatment had a significantly higher probability of being unemployed at several time points and had higher lifetime unemployment. This supports recent research demonstrating the "long shadow" of childhood psychiatric problems on adult income and well-being (Goodman et al., 2011).

DOI: 10.1177/0956797615569001

Table S5. Extended unemployment regressions for NCDS for the period 1974-82.

Unemployment	Age 23 [a]	Age 33 [a]	Age 42 [a]	Age 50 [a]	Lifetime [b]
N	4,207	3,840	4,030	3,547	5,383
Self-Control [c]	-0.023***	-0.012***	-0.007***	-0.003	-0.172***
	(0.005)	(0.003)	(0.002)	(0.002)	(0.050)
Intelligence [c]	-0.012**	-0.010***	-0.004	-0.005*	-0.126***
	(0.005)	(0.004)	(0.003)	(0.003)	(0.046)
Female	0.004	-0.017**	-0.005	-0.012**	-0.449***
	(0.009)	(0.007)	(0.005)	(0.005)	(0.082)
Social class (base = I) [d]					
II	0.004	-0.038**	0.006	-0.004	-0.204
	(0.024)	(0.018)	(0.008)	(0.013)	(0.203)
III	0.004	-0.009	0.011	-0.000	-0.067
	(0.022)	(0.015)	(0.007)	(0.012)	(0.188)
IV	-0.003	-0.012	0.012	-0.007	0.130
	(0.025)	(0.017)	(0.009)	(0.013)	(0.217)
V	0.038	0.015	0.028**	-0.003	0.310
	(0.028)	(0.018)	(0.013)	(0.014)	(0.236)
Childhood Experiences					
Family Difficulties Scale	0.003	-0.002	-0.001	0.007*	0.098
	(0.009)	(0.006)	(0.004)	(0.004)	(0.077)
Low birth weight	0.011	0.011	-0.002	0.014	0.029
	(0.021)	(0.015)	(0.011)	(0.009)	(0.190)
Psychiatric problems	0.065***	0.027	0.000	0.021**	0.430*
	(0.023)	(0.016)	(0.012)	(0.011)	(0.249)
Headaches or Epilepsy	0.030**	0.003	0.001	0.002	0.263*
	(0.014)	(0.011)	(0.007)	(0.008)	(0.139)
Mental Retardation	0.029	0.035	0.018	0.015	0.405
	(0.031)	(0.022)	(0.014)	(0.015)	(0.341)
Region of birth (base=North)					
North West	-0.020	0.009	0.008	0.012	0.168
	(0.022)	(0.013)	(0.011)	(0.013)	(0.172)
East-West Riding	-0.045*	0.006	-0.009	-0.020**	0.020
	(0.024)	(0.014)	(0.010)	(0.010)	(0.190)
North Middle	-0.077***	-0.006	-0.008	0.002	-0.280
	(0.021)	(0.015)	(0.010)	(0.013)	(0.189)
Middle	-0.031	-0.000	-0.001	-0.000	0.232
	(0.023)	(0.014)	(0.010)	(0.012)	(0.179)
East	-0.080***	-0.033*	-0.006	-0.001	-0.307
	(0.021)	(0.018)	(0.010)	(0.012)	(0.191)
South East	-0.073***	-0.011	0.002	-0.006	-0.210
	(0.020)	(0.013)	(0.010)	(0.010)	(0.165)
South	-0.079***	-0.039*	-0.012	-0.009	-0.535**
	(0.023)	(0.021)	(0.010)	(0.012)	(0.209)
South West	-0.062***	-0.016	-0.017**	-0.020**	-0.164
	(0.023)	(0.017)	(0.009)	(0.010)	(0.200)
Wales	0.013	0.003	-0.005	-0.009	0.049
	(0.028)	(0.016)	(0.011)	(0.012)	(0.209)

Scotland	0.006	-0.010	0.005	-0.008	0.118
	(0.023)	(0.014)	(0.010)	(0.010)	(0.171)
Demographics					
Husband age at birth	-0.000	0.001*	0.000	0.000	0.007
	(0.001)	(0.001)	(0.000)	(0.000)	(0.006)
Household Size	0.009***	0.005**	0.001	0.001	0.116***
	(0.003)	(0.002)	(0.002)	(0.002)	(0.031)
Race	0.105**	0.013	-0.004	0.038**	-0.334
	(0.042)	(0.027)	(0.023)	(0.017)	(0.435)
Length of activity history					0.003***
					(0.000)

[a] Regressions contain Probit marginal effect coefficients.
[b] Regression contains negative binomial coefficients.
[c] Self-Control and intelligence are standardized.
[d] Social class was derived from the father's occupation: I = higher administration, II = managerial or technical occupations, III = skilled workers, IV = semiskilled workers, and V = unskilled workers. Social class I was the reference group.
Standard errors in parentheses
*** p<0.01, ** p<0.05, * p<0.1

Section 6: Unemployment regressions controlling for prior unemployment in Study 1 and 2.

The regressions in Table S6 control for employment status at previous time-points. In Study 1 this means we estimated the probability of unemployment at age 30, 34, 38 and 42 while controlling for pripr unemployment. In Study 2 we predicted the probability of unemployment at age 33, 42 and 50 while controlling for prior unemployment. In both studies better self-control predicted a reduced probability of unemployment even after controlling for prior unemployment, although the coefficients decreased by 45% on average. Nonetheless the coefficients remain significant at p<0.01, indicating that poor childhood self-control has a strong association with later unemployment above and beyond the contributing effects of intermediating unemployment.

Table S6. Unemployment regressions for the British Cohort Study (Study 1) and National Child Development Study (Study 2), controlling for prior unemployment.

Observations	19,768	15,391	20,436	16,398
Study	1	1	2	2
Self-Control	-0.008***	-0.004***	-0.010***	-0.006***
	(0.001)	(0.001)	(0.002)	(0.001)
Unemployment (t-1)		0.060***		0.051***
		(0.004)		(0.004)

All columns report Probit marginal effects coefficients controlling for childhood intelligence, gender, social class and a time trend.

DOI: 10.1177/0956797615569001

Robust standard errors in parentheses.
*** $p<0.01$

DOI: 10.1177/0956797615569001

References

Barkley, R. (1997). *ADHD and the Nature of Self-control*. New York: Guilford Press.

Conners, C. (1969). A teacher rating scale for use in drug studies with children. *American Journal of Psychiatry*, *126,* 884–888.

Currie, J. & Widom, C.S. (2010). Long-term Consequences of Child Abuse and Neglect on Adult Economic Well-being. *Child Maltreatment*, 15, 111–1120.

Duckworth, A. L. & Seligman, M. E. P. (2005). Self-discipline outdoes IQ predicting academic performance in adolescents. *Psychological Science, 16,* 939–944.

Duckworth, A. L. & Seligman, M. E. P. (2006). Self-discipline gives girls the edge: Gender differences in self-discipline, grades, and achievement test scores. *Journal of Educational Psychology, 98*, 198–208.

Duckworth, A. L., Tsukayama, E., & Kirby, T. (2013). Is it really self-control? Examining the predictive power of the delay of gratification test. *Personality and Social Psychology Bulletin, 39*, 843–855.

Goodman A., Joyce R. & Smith J. (2011). The long shadow cast by childhood physical and mental problems on adult life. *Proceedings of the National Academy of Science*, 108, 6032–6037.

Goodman, R. (1997). The Strengths and Difficulties Questionnaire: a research note. *Journal of child psychology and psychiatry*, 38, 581–586.

Liu, Y., Croft, J., Chapman, D., Perry, G., Greenlund, K., Zhao, G. & Edwards, V. (2013). Relationship between adverse childhood experiences and unemployment among adults from five U.S. states. *Social Psychiatry and Psychiatric Epidemiology*, 48, 357–369.

Kawakami, N., Abdulghani, E.A. Alonso, J., Bromet, E. J., Bruffaerts, R., Caldas-De-Almeida, J. M.… Kessler, R. C. (2012). Early-Life Mental Disorders and Adult Household Income in the World Mental Health Surveys. *Biological Psychiatry*, 72, 228–237.

Rutter, M. (1967). A children's behaviour questionnaire for completion by teachers: preliminary findings. *Journal of Child Psychology and Psychiatry*, 8, 1-11.

Stott, D. H. (1969). *The social-adjustment of children: Manual to the Bristol social-adjustment guides.* London: University of London Press.

Tangney, J., Baumeister, R. and Boone, A. (2004). High self-control predicts good adjustment, less pathology, better grades, and interpersonal success. *Journal of personality*, 72, 271–324.

Tsukayama, E., Duckworth, A. L., & Kim, B. E. (2013). Domain-specific impulsivity in school-age children. *Developmental Science, 16*, 879-893.
